# Tunable Wettability Pattern Transfer Photothermally Achieved on Zinc with Microholes Fabricated by Femtosecond Laser

**DOI:** 10.3390/mi12050547

**Published:** 2021-05-11

**Authors:** Fengping Li, Guang Feng, Xiaojun Yang, Chengji Lu, Guang Ma, Xiaogang Li, Wei Xue, Haoran Sun

**Affiliations:** 1 School of Aerospace Engineering, Xiamen University, Xiamen 361005, China; 2 Zhejiang Provincial Engineering Lab of Laser and Optoelectronic Intelligent Manufacturing, Wenzhou University, Wenzhou 325035, China; fengguang0150@link.tyut.edu.cn (G.F.); lcj@wzu.edu.cn (C.L.); ma_guang@wzu.edu.cn (G.M.); lixiaogang@lyncwell.cn (X.L.); xw@wzu.edu.cn (W.X.); sun13736712970@163.com (H.S.); 3 Xi’an Institute of Optics and Precision Mechanics, Chinese Academy of Sciences, Xi’an 710119, China; laser_ceo@opt.cn

**Keywords:** tunable wettability, wettability pattern, femtosecond laser

## Abstract

A quickly tunable wettability pattern plays an important role in regulating the surface behavior of liquids. Light irradiation can effectively control the pattern to achieve a specific wettability pattern on the photoresponsive material. However, metal oxide materials based on light adjustable wettability have a low regulation efficiency. In this paper, zinc (Zn) superhydrophobic surfaces can be obtained by femtosecond-laser-ablated microholes. Owing to ultraviolet (UV) irradiation increasing the surface energy of Zn and heating water temperature decreasing the surface energy of water, the wettability of Zn can be quickly tuned photothermally. Then, the Zn superhydrophobic surfaces can be restored by heating in the dark. Moreover, by tuning the pattern of UV irradiation, a specific wettability pattern can be transferred by the Zn microholes, which has a potential application value in the field of new location-controlled micro-/nanofluidic devices, such as microreactors and lab-on-chip devices.

## 1. Introduction

In nature, the lotus has excellent superhydrophobic surfaces [1]. After long-term research, it has been determined that in the characterization of superhydrophobic surfaces, they must have a certain rough structure and low surface energy [2]. Therefore, there are two methods for preparing superhydrophobic surfaces: one is fabricating a rough structure with low surface energy; the other is fabricating a rough surface and then spraying a low-surface-energy modifier onto it. However, the fixed wettability surface cannot satisfy the needs of a functional surface. Tunable wettability surfaces play an important role in regulating the surface behavior of liquids, which can be used for printing [3], droplet transfer [4], microfluidic systems [5], oil-water separation [6,7], and underwater gas collection [8,9,10].

In recent years, the tunable wettability of some metal oxide materials under light stimulation has attracted much attention [11,12,13]. The metal oxide ZnO, which is an important light-sensitive material, has the advantages of good stability and low cost, but its regulation efficiency is low. For example, Yong et al. [14] used a femtosecond laser to ablate cross micro-/nanostructures on the surface of a zinc (Zn) plate, and realized the regulation between superhydrophobic and superhydrophilic surfaces in ultraviolet (UV) and dark environments. However, the superhydrophobic Zn surface must be irradiated by UV light for 24 h to become a quasi-superhydrophilic surface, and then the quasi-superhydrophilic surface must be placed in a dark environment for 7 d to become a superhydrophobic surface. Tian et al. [15] used UV light to irradiate a stainless-steel mesh covered with ZnO nanorods to achieve regulation between superhydrophobic and superhydrophilic surfaces. However, the regulation efficiency was also low. To improve the efficiency of wettability regulation, Bai et al. [16] used stearic acid ethanol solution and sodium hydroxide to quickly adjust the wettability of stainless steel coated with nano Zn oxide. The regulation can be quickly switched in 15 min, but this method requires two kinds of chemical reagents to tune the wettability and does not have the ability to form a wettability pattern.

At the same time, the temperature can also be used to adjust the wettability. For example, Liu et al. [17] realized the rapid regulation between superhydrophobic and superhydrophilic aluminum (Al) surfaces by adjusting the temperature of the Al surface and the pressure between the water and Al, and still achieved excellent recoverability, stability, and repeatability after 10 cycles. Moreover, Ngo et al. [18] achieved the adjustment of wettability by heating copper, Al, and titanium at different temperatures. The higher the temperature, the shorter the conversion time from superhydrophilic to superhydrophobic. However, this method does not have the ability to form a wettability pattern. Furthermore, tunable wettability can be achieved by changing the water temperature [19]. Liu et al. [20] investigated the repellent hot water of superhydrophobic surfaces and found that the superhydrophobic surfaces usually exhibited a high repellency to cool water. However, such surfaces show a remarkably decreased repellency to hot water, which can be attributed to a decrease in the surface tension. Moreover, the surface structures can be destroyed by elevated temperatures [21].

Meanwhile, the magnetic field [22], electric field [23], and mechanical force [24] have been commonly used to achieve tunable wettability. For example, Tian et al. [25] used ferromagnetic micro-nanomaterials to prepare a magnetic fluid/ZnO nanoarray. A water droplet could follow the motion of the gradient composite interface structure as it responded to the gradient magnetic field motion, which achieved tunable wettability. In addition, Tian et al. [26] further proposed a method for photoelectric coordination to achieve tunable wettability. The photosensitizer material titanium phthalocyanine was coated on the ZnO nanorods, and the structure was modified by heptafluorododecyltrimethoxysilane. The capillary pressure between the micro/nanostructures is tuned by a voltage combined with the light-modified photosensitizer in order to tune the wettability. Yang et al. [27] used a femtosecond laser to prepare the superhydrophobic surface of silicone rubber. The superhydrophobic surface of silicone rubber was transformed from the “petal” state to the “lotus” state by stretching.

Compared with light and thermal tune wettability, the advantage of the magnetic field is that it can quickly achieve tunable wettability, but the disadvantage is that magnetic materials must be added to soft materials, which limits the scope of application. The voltage can quickly tune wettability, but the material must be conductive. The shortcomings of the mechanical force are also due to the limitations of the material. This method can only use materials with flexibility and high ductility to achieve tunable wettability.

The above methods mainly serve to control the properties of materials in order to achieve tunable wettability, which is easily affected by the defects of material properties. At present, optical, magnetic, electrical, mechanical forces and other factors are seldom combined with liquid temperature to achieve tunable wettability. Because the temperature of the liquid is not affected by the material properties, it can not only improve the efficiency of tunable wettability but also provide a new idea for achieving tunable wettability. Therefore, to achieve a quickly tunable wettability pattern, in the work described in this paper, a femtosecond laser was used to fabricate microholes on Zn foil placed in the dark and heated at 100 °C for 12 h to obtain a superhydrophobic surface. The wettability pattern was then able to be obtained by controlling the pattern of UV irradiation. Moreover, with an increasing water temperature, the surface tension of water decreases. Therefore, the wettability pattern of the Zn-foil surface can be quickly transferred to paper through microholes. This method has potential applications in new location-controlled micro-/nanofluidic devices, such as microreactors and lab-on-chip devices.

## 2. Sample Fabrication

The size of the Zn foil sample measured 20 × 20 × 0.04 mm^3^. A Light Conversion L17771 femtosecond laser was used, which emitted a wavelength of 1064 nm with a maximum average power of 20 W, a pulse width of 200 fs, and a focusing spot diameter of 20 μm. Before the sample was ablated by the femtosecond laser, the sample was cleaned with an ultrasonic cleaning machine under alcohol for 10 min to remove surface impurities and was then taken out to dry. The specific laser parameters were the following: the diameter of the microhole was 20, 30, 40, 50, 60, and 70 μm, respectively, and the distance between the two microholes was 100 μm. The laser scanning speed was 2000 mm/s, the power was 10 W, and the frequency was 100 kHz.

## 3. Results

**Figure 1:**
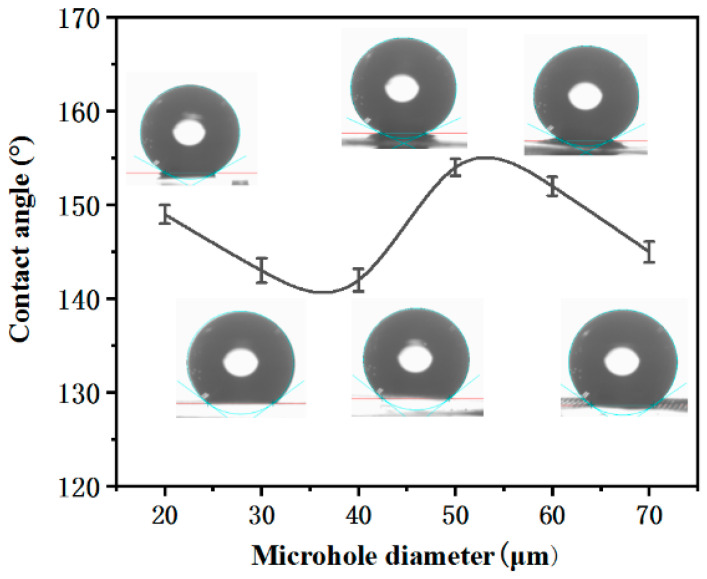
The contact angle of microholes with different diameters.

Figure 1 shows the contact angles (CA) of the microhole with diameters of 20, 30, 40, 50, 60, and 70 μm, respectively. The CA increases with the increase of the microhole diameter. When the microhole diameter is 50 μm, the CA reaches the maximum. With the further increase of the microhole diameter, the CA will decrease, which may be due to the lesser microstructure for supporting liquid with the increase of the microhole diameter. However, when the diameter of the microhole is 20 μm, the CA is larger than that of 30 μm and 40 μm, which is because more areas that are not ablated are coated by the microfragments. Therefore, the sample with a microhole diameter of 50 μm is researched in this paper.

**Figure 2:**
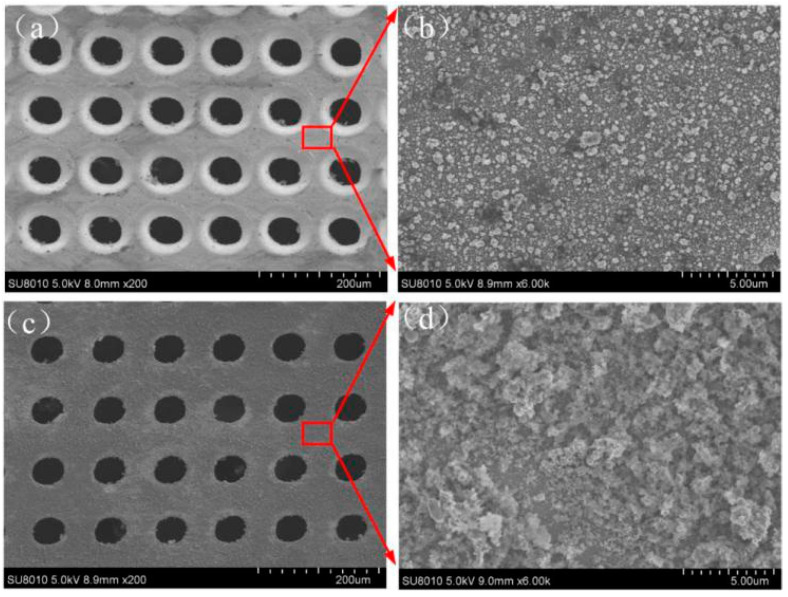
SEM of Zn after femtosecond-laser ablation. (**a**) Entrance of microhole. (**c**) The exit of the microhole. (**b**,**d**) are the local amplifications of (**a**,**c**), respectively.

**Figure 3:**
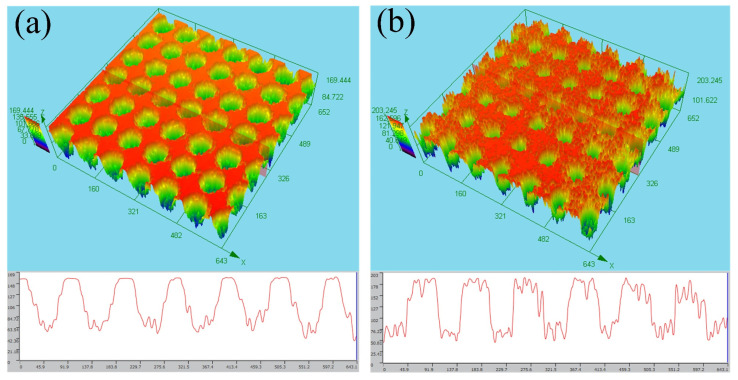
3D image profile of the femtosecond-laser-ablated surface. (**a**,**b**) are the entrances and exits of the microholes, respectively.

Scanning-electron-microscopy (SEM) images of the microholes with a diameter of 50 μm ablated by the femtosecond laser are shown in Figure 2. The entrance and exit of the microhole are shown in Figure 2a,c, respectively, and the local amplification is shown in Figure 2b,d, respectively. Owing to the ultrashort pulse of the femtosecond laser having no obvious thermal effect, the surface of the Zn foil has a small layer, but the micro-fragments shown in Figure 2b,d are formed. The sizes of the microfragments range from the nano- to microscales. These microfragments and microholes form the micro-/nanostructure on the surface of the Zn foil, which is the one of the keys to the formation of a superhydrophobic surface. Meanwhile, the three-dimensional (3D) image profile of the femtosecond-laser-ablated surface is shown in Figure 3. The entrance and exit of the microholes are shown in Figure 3a,b respectively.

**Figure 4:**
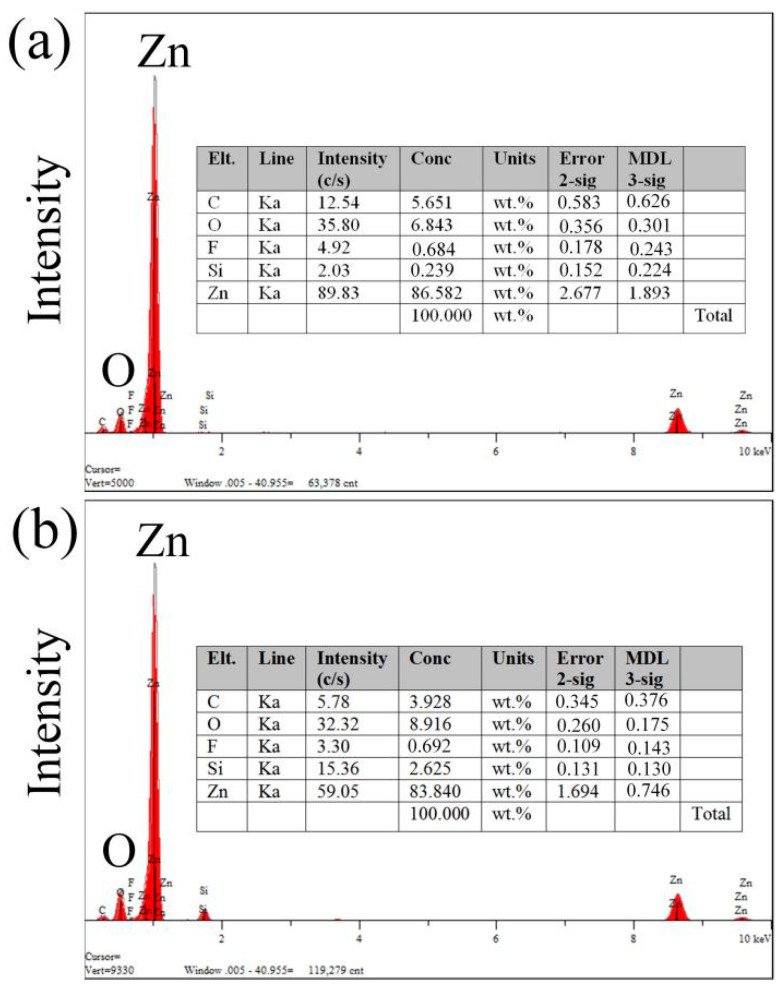
Weight percentages of different elements after femtosecond-laser ablation. (**a**) Entrances and (**b**) exits of the microholes.

Figure 4 shows the weight percentages of different elements after the femtosecond-laser ablation, which were investigated by X-ray spectroscopy (EDXS). Elemental C, O, and Zn exhibit obvious changes. Owing to the Zn forming a rough ZnO layer on the surface after the femtosecond-laser ablation, the weight percentage of elemental O increased from 0% to 6.843% and 8.916% and the weight percentage of elemental Zn decreased from 100% to 86.582% and 83.840%. The increase of elemental O resulted in the decrease of the surface energy of Zn, which is another crucial factor in the formation of a superhydrophobic surface. However, owing to the fact that the Zn was ablated on the glass substrate, the F and Si of glass could be deposited on the surface of Zn.

**Figure 5:**
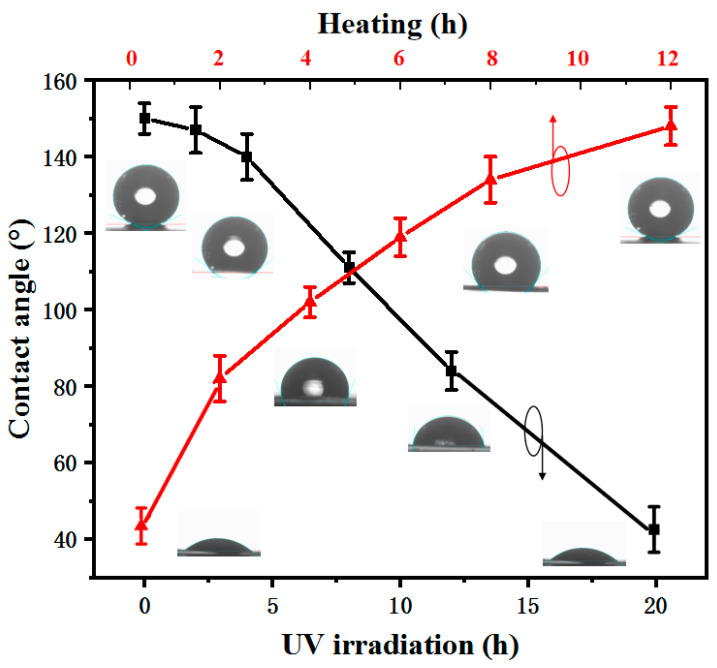
CA curve of Zn with variations of heating and UV irradiation.

The wettability transformation between superhydrophobicity and hydrophobicity can be achieved by UV irradiation and heating in the dark, as shown in Figure 5. With an increasing UV-irradiation time, the CA of the black curve gradually decreases, and the wettability of the sample changes from a superhydrophobic surface to a hydrophilic surface. The UV power was 36 W; the higher the UV power, the shorter the UV-irradiation time. Therefore, the time of the wettability transformation can also be decreased by increasing the UV power [3]. As is well known [28,29,30], UV irradiation produces electron-hole pairs in the ZnO lattice. The electrons and holes produced under UV irradiation will move to the surface, and water and O in the air may competitively adsorb on these vacancies. Compared with O adsorption, Zn^2+^ defect sites are more favorable for hydroxyl adsorption. Finally, the UV-irradiated surface will form functional groups with hydroxyl groups, resulting in the hydrophilicity of the Zn foil surface.

Furthermore, the superhydrophobic surface can be restored by heating in the dark. As shown in Figure 5, with an increasing heating time in the dark environment, the CA of the red curve is larger. The heating time determines the hydrophobicity of the Zn foil surface. The Zn surface under UV irradiation is hydrophilic, and the CA is 42°. Finally, after heating in the dark for 12 h, the superhydrophobic surface is obtained, and the CA is 152°. The main reason for the wettability transformation is that the O atoms in the air can gradually replace hydroxyl during heating in the dark environment, and the wettability is restored from hydrophilicity to superhydrophobicity.

The wettability transformation of the Zn surface between a superhydrophobic surface and a hydrophilic one can be tuned by UV irradiation and heating in the dark. The pattern of UV irradiation is controllable, but the time of the wettability transformation becomes very long. Therefore, a quickly tunable wettability and wettability pattern can be achieved by UV irradiation and increasing the water temperature.

**Figure 6:**
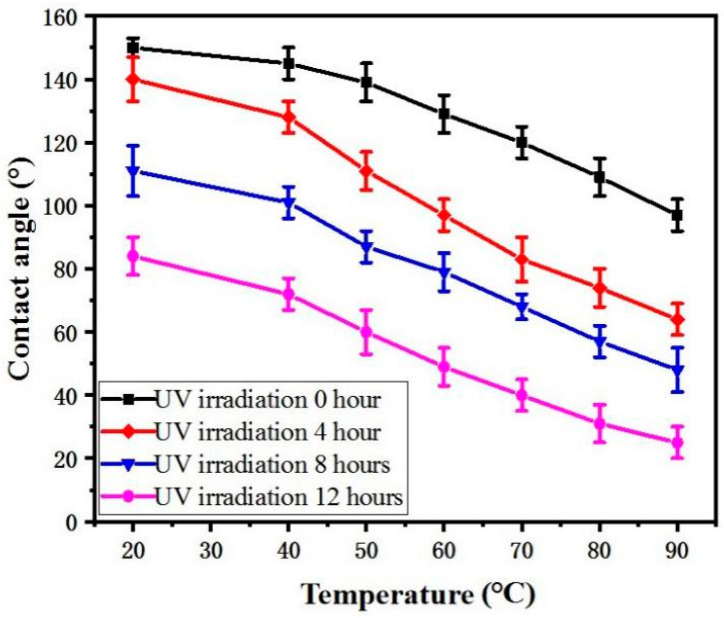
CA curve of Zn with variations of UV irradiation and water temperature.

As shown in Figure 6, the influence of UV irradiation and water temperature on wettability was investigated. When the UV irradiation time was 0, 4, 8, and 12 h, the CA of the Zn surface decreased. With an increasing water temperature, the Zn surface changed from a superhydrophobic/hydrophobic to a hydrophilic surface. This can be attributed to a decrease in surface tension when the water temperature increases, and the lower surface tension of hot water makes it a better “wetting agent” for penetrating into the microstructure of rough surfaces rather than bridging them with surface tension [20]. When 90 °C water was dropped onto the Zn surface under no UV irradiation, the CA of the Zn surface was 100°. This surface can then be called a hydrophobic surface. When 70 °C or higher water was dropped onto the Zn surface under UV irradiation for 4 h, the CA of the Zn surface was below 90° and was thus a hydrophilic surface. When the Zn surface was under UV irradiation for 8 h, water above −50 °C could achieve a hydrophilic effect. However, When the Zn surface was under UV irradiation for 12 h, it was hydrophilic at any temperature, and the minimum CA of the Zn was 28°.

If the water temperature is not considered, the wettability transformation can be achieved by using UV light alone, but it requires at least 20 h. Therefore, the time can be reduced to 8 h by increasing the water temperature. When the UV-irradiation time is only 8 h, the time it takes for the sample to recover the superhydrophobic surface by heating in the dark can be reduced to 6 h.

Therefore, the wettability transformation from a superhydrophobic to hydrophilic surface can be used to realize a quickly tunable wettability pattern. However, when the water temperature is over 50 °C, the surface tension of water decreases and the water molecules move faster, resulting in the water expanding quickly. Thus, a clear wettability pattern will not be obtained when water droplets are transferred to the paper through microholes.

**Figure 7:**
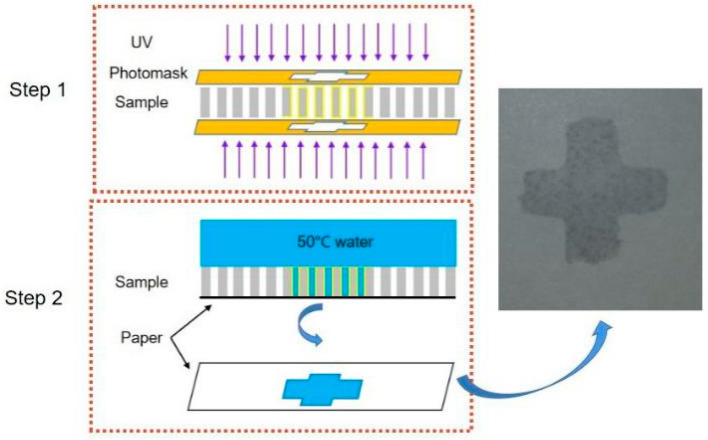
Specific wettability pattern using UV irradiation and water temperature.

As shown in Figure 7, the first step is to irradiate the Zn superhydrophobic surface with UV light for 8 h; the UV light irradiates the obverse and reverse of the Zn foil by a mask plate with a cross-pattern. The second step is to drop 50 °C water onto the upper surface of the Zn foil, place a piece of paper on its lower surface, and then gently press the Zn foil against the paper. The result is that the water will penetrate through the microholes in the area that is UV-irradiated. Finally, the wetted cross-pattern can be observed on the paper, as shown in Figure 7.

To explain the above phenomenon, the regulation mechanism of wettability must be understood. The wettability transformation is mainly done to tune the surface energy between the water and solid. The surface energy of water is higher than metal at room temperature and atmospheric pressure. Therefore, the metal surface is generally hydrophilic. The roughening of the metal surface is obtained by laser ablation, and then the oxide layer is formed on the rough surface by heating in the dark, which decreases the surface energy of the metal surface. Therefore, the differentials between the surface energy of the metal surface and that of water are increased, resulting in a strong repulsive force between the water and metal surface, and, thus, a superhydrophobic surface is finally formed.

**Figure 8:**
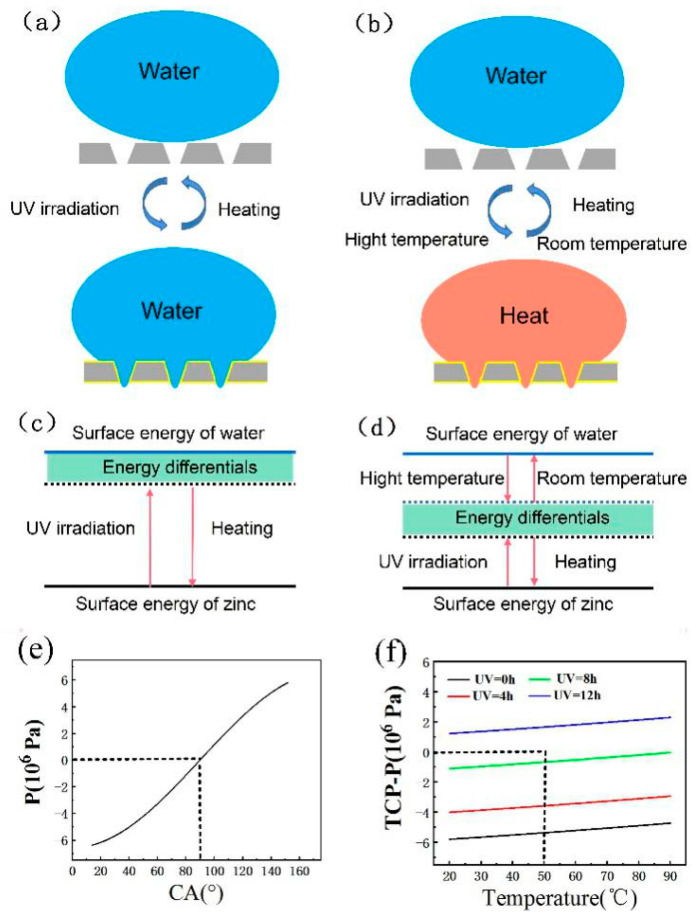
(**a**,**c**) Mechanism of UV irradiation adjusting the wettability transformation. (**b**,**d**) Mechanism of UV irradiation and water temperature adjusting the wettability transformation. (**e**) Pressure of the water with different CA. (**f**) Temperature conducting pressure of the water with different temperature.

As shown in Figure 8a,c, the wettability of the Zn surface is mainly tuned by UV irradiation of the superhydrophobic Zn foil surface, which results in the formation of hydroxyl groups. Therefore, the surface energy of the Zn foil will increase after UV irradiation. However, the surface of the Zn foil is hydrophilic, and therefore there is a differential between water and Zn. However, the small surface differentials cannot prevent water droplets from wetting the Zn foil and penetrating the microholes. The hydrophilic surface can be heated in the dark, and then the superhydrophobic surface can be obtained. However, this method has an obvious disadvantage in that the time of the wettability transformation is very long. The surface energy of the Zn foil’s superhydrophobic surface increases after UV irradiation and then returns to the low-surface-superhydrophobicity surface by heating in the dark environment. In this process, only the surface energy of the Zn foil is constantly adjusted. Therefore, the process is inefficient.

To improve the wettability transformation efficiency of the Zn foil, the water temperature is considered herein, and the surface energy of water can be adjusted by temperature. As shown in Figure 8b,d, first the surface energy of the Zn foil is increased by UV irradiation, and the surface energy of water is decreased by increasing the water temperature. The differentials in surface energy between water and Zn can quickly decrease. Therefore, the superhydrophobic Zn foil surface is quickly transformed into a hydrophilic surface. The time to heat the water is very short, negligible compared with the UV-irradiation time. Therefore, this method can greatly improve the efficiency of the wettability transformation. Moreover, because the Zn superhydrophobic surface is formed by its chemical transformation, the increasing temperature of hot water will not damage the chemical composition of the surface. When the water temperature returns to room temperature and the Zn is placed in the dark and heated for some time, the surface of the Zn foil can quickly return to a superhydrophobic surface.

Upon comparing Figure 8b,d with Figure 8a,c, it can be seen that the wettability transformation time between the superhydrophobic and hydrophilic surfaces is significantly reduced. Therefore, the controllable pattern of UV irradiation and water temperature on the surface energy of water can be combined to achieve a quickly tunable wettability pattern.

The mechanism of the wettability transformation can be expressed by the following equations [3,31]:

*Γ(T*) = 75.714 − 0.1414 × *T* − 2.5399 × 10^−4^ × *T*^2^ (20 °C ≤ *T* ≤ 90 °C) (1)

*P =* −*l* × *Γ* × *cosθ*/*A* (0° ≤ *T* ≤ 180°) (2)

*TCP* = *l* × Δ*Γ*/*A* (3)

where *Γ* is the surface tension of the liquid–vapor interface, *T* is the temperature of water, *P* is the hydrostatic pressure, *l* is the circumference of the microholes, *θ* is the CA, *A* is the cross-sectional area of the microhole, and *TCP* is the temperature conducting pressure.

*P* = 5.80 × 10^6^ Pa—which is a considerable wetting energy barrier at atmospheric pressure—must be overcome for the liquid to penetrate into the microholes. According to Equation (2), the decrease of *θ* will lead to the decrease of *P*, as shown in Figure 8e. When the CA is lower than 90°, *P* is lower than zero. Therefore, the UV irradiation can decrease *θ*, which can cause the wettability transformation from the superhydrophobic to hydrophilic surface. In addition, the water temperature can increase the *TCP*, and when *TCP*−*P* > 0, the liquid can penetrate the microholes, as shown in Figure 8f. With an increasing UV-irradiation time and temperature, the *TCP*−*P* value gradually increases. However, when the UV-irradiation time is 8 h and the water temperature is 50 °C, *TCP*−*P* is not zero, which differs from the experiment to some degree. When the UV-irradiation time is 12 h, the value of *TCP*−*P* is larger than zero at any temperature. Therefore, by increasing the water temperature and UV irradiation time at the same time, the wettability transition can be quickly realized.

## 4. Conclusions

In summary, a Zn superhydrophobic surface can be achieved by femtosecond-laser-ablated microholes. The Zn superhydrophobic surface can be quickly transformed into a hydrophilic surface by adjusting both the UV irradiation and water temperature. UV irradiation can control the wettability pattern of the Zn surface, while the hot water can decrease the surface tension of water to obtain a low surface energy of water. Therefore, the specific wettability pattern can be transferred through microholes in Zn foil. Compared with the adjustment of the UV radiation or temperature alone, the proposed method can effectively improve the wettability-transformation efficiency and achieve specific wettability patterns. Therefore, the method has good potential application prospects in new location-controlled micro-/nanofluidic devices, such as microreactors and lab-on-chip devices, among others.

## Data Availability

The datasets are available from the corresponding author upon reasonable request.
